# Sex Differences in the Processing of Global vs. Local Stimulus Aspects in a Two-Digit Number Comparison Task – An fMRI Study

**DOI:** 10.1371/journal.pone.0053824

**Published:** 2013-01-15

**Authors:** Belinda Pletzer, Martin Kronbichler, Hans-Christoph Nuerk, Hubert Kerschbaum

**Affiliations:** 1 Department of Cell Biology, University of Salzburg, Salzburg, Austria; 2 Department of Psychology, University of Salzburg, Salzburg, Austria; 3 Center for Neurocognitive Research, University of Salzburg, Salzburg, Austria; 4 Department of Neurobiology & Behavior, University of California Irvine, Irvine, United States of America; 5 Neuroscience Institute & Department of Neurology, Christian Doppler Clinic, Paracelsus Medical University, Salzburg, Austria; 6 Department of Psychology, University of Tuebingen & Knowledge Media Research Center, Tuebingen, Germany; University of British Columbia, Canada

## Abstract

It has been debated for several decades, whether number magnitudes are processed global/holistically (whole number magnitudes) or in a local/decomposed fashion (digit magnitudes). However, while it has been suggested that men attend stronger to the global level, while women attend stronger to the local level, the question has never been studied with regards to sex differences. In two-digit number comparison men should engage a more holistic processing strategy, while women should engage a more decomposed strategy. To test this hypothesis, we employed number comparison stimuli of varying decade crossing and unit-decade compatibility in men (n = 16) and women (n = 16) during their early follicular and mid-luteal cycle phase. In within-decade (WD) items both numbers had the same decade digits. Non-WD items were unit-decade-compatible, if the smaller number contained the smaller unit-digit and incompatible otherwise. In incompatible items the two local features require different responses. Thus, processing of the local level should result in a compatibility effect in RT and recruitment of differential neural networks for compatible and incompatible items. The results support the view of a holistic strategy in men and a decomposed strategy in women. In men RT and BOLD-response did not differ for incompatible compared to compatible items. Women respond slower to incompatible compared to compatible items. They show a BOLD-response compatibility effect in regions of the default mode network during their follicular phase and in prefrontal areas involved in inhibitory control during their luteal phase. Furthermore, lateralization indices interacted with decade crossing and menstrual cycle phase in a way consistent with the hypothesis of progesterone-mediated interhemispheric decoupling.

## Introduction

Sex-dependent behavioural differences have been observed in a variety of cognitive tasks (see [1] for a review) and associated with differences in brain structure and function [2]–[4]. Although several of these differences have been attributed to the use of different cognitive strategies, general (visual) attentional processes preceding the task-specific cognitive processing component have widely been neglected in the sex-difference literature. Most stimuli are hierarchical, with global structures being made up of local parts and it seems to be an individual characteristic, whether a more global or local processing style is employed [5]. The small number of studies addressing the issue of sex differences in global and local processing support the view that men use a more global strategy in visual processing, while women have a stronger focus on the local level [6]–[9]. While responses to the global level are faster than responses to the local level (global advantage) in men [9], responses to the local level are faster than responses to the global level (local advantage) in women [7]. This dissociation is in line, although it has not directly been studied, with sex-specific strategy use reported from other cognitive tasks. For example in spatial navigation men use more allocentric ( = global) landmarks and women more egocentric ( = local) landmarks [10]–[12]. Similar evidence comes from emotional memory tasks [13]–[14]. After watching an emotional story, men are better at recalling the gist of the story, while women are better at recalling details. Furthermore, recent evidence from the emotional memory task suggests, that the processing style of women depends on their hormonal status. There seems to be a shift from local to global processing during the low hormone follicular phase and in women on hormonal contraceptives [15]. However, hormonal status has not been taken into account in other studies on sex differences in global and local processing.

Early works suggest that global processing relies on the right hemisphere, while local processing involves the left hemisphere [16]–[17]. However, more recent results question this view and state that hemispheric asymmetries in global-local tasks depend on the content or stimulus material used [18]–[19]. When subjects were presented with hierarchical stimuli composed of objects and shapes rather than letters, processing of the global level was associated with activation in the left hemisphere and processing of the local level with activation in the right hemisphere [18]. Fink and colleagues propose the following model to explain their results: both hemispheres are by default in global mode and the more demanding local information is processed in the hemisphere specialized for the content. Importantly however, all their results have been obtained in male participants only. Women’s hemispheres may not by default be in global mode but in local mode and for women global information may be more demanding than local information. Several studies suggest that hemispheric asymmetries, e.g. for verbal and spatial content, is less pronounced in women than in men and that women show stronger interhemispheric connectivity [20]. This may well relate to less segregated processing of global and local information in women. Interhemispheric interactions are furthermore subject to menstrual cycle dependent changes [21]–[22] with progesterone mediating decoupling between the hemispheres. It has been demonstrated that the global-local dissociation between the sexes in emotional tasks and hierarchical letter stimuli is accompanied by stronger left-lateralized processing in women and stronger right-lateralized processing in men [7], [9], [14].

The present study seeks to apply these findings to point a years-long discussion in the number processing literature in a new direction. For several decades it has been debated whether the magnitude of multi-digit numbers is processed in a holistic (magnitude of whole number) or decomposed (magnitude of digits) fashion [23]–[24] However, while this question clearly translates to the processing of global vs. local aspects of numerical stimuli, the evidence pointing in one direction or the other has not been addressed with respect to sex differences, menstrual cycle or hemispheric asymmetries. Based on the results discussed above, we predict a more global/holistic processing strategy in men and a more local/decomposed processing strategy in women, which may furthermore be subject to menstrual cycle dependent changes.

Behavioural studies indicate that number magnitudes have a spatial representation, with smaller numbers represented to the left side of space and larger numbers to the right. This representation is metaphorically referred to as the mental number line. Patient and neuroimaging studies indicate that numerical-spatial interactions arise from common parietal circuits for spatial and numerical processing (for reviews see [25], [26]). Specifically, the intraparietal sulcus (IPS) emerged as the common activation site of several number magnitude tasks on the one hand [25] and spatial tasks on the other hand [27]. Furthermore, the posterior superior parietal lobule (PSPL) emerges as a common activation site during attentional orientation in external space and internal attentional orientation on the mental number line, as for example during numerical comparison [25], [28].

**Figure 1:**
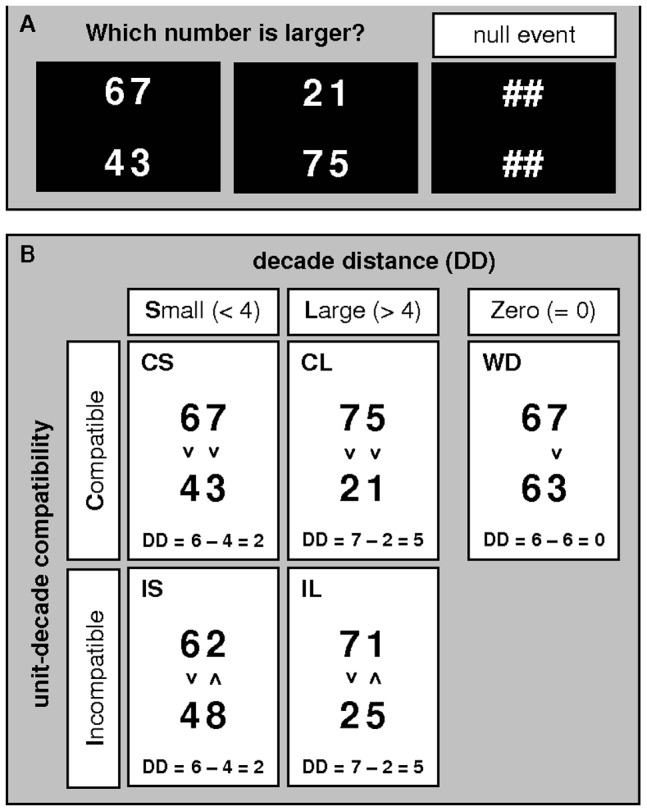
Task. A. Examples of number comparison items. Left: the upper number is larger, middle: the lower number is larger, right: null event (NE). B. Examples of (i) compatible items with small decade distance (CS), (ii) compatible items with large decade distance (CL), (iii) incompatible items with small decade distance (IS), (iv) incompatible items with large decade distance (IL) and (v) within-decade items (WD). DD = decade distance.

To assess sex differences and menstrual cycle dependent changes in holistic and decomposed strategy use and relate those to hemispheric asymmetries, we employ a two-digit number comparison task during functional MRI between men and women during different cycle phases. Participants have to decide, which of two vertically aligned two-digit numbers is larger. This task allows for two manipulations (compare Figure 1) demonstrating whether participants base their decisions on the whole numbers (global/holistic processing) versus the digits (local/decomposed processing). On the one hand, we assess the effect of *decade crossing* by comparing within-decade (WD) items (e.g. 68_63), in which both numbers contain the same decade digit (decade distance = 0), to non-WD items, in which the two numbers contain different decade digits (decade distance >0). While on the local level the comparison of non-WD items can be based on decade digits only, the comparison of WD items requires to processing of both decade and unit digits. On the other hand, we assess the effect of compatibility within non-WD items by comparing unit-decade compatible items, in which the larger number contains the larger unit digit (68_23) to unit-decade incompatible items (63_28), in which the larger number contains the smaller unit digit. While in compatible items, all item features (magnitude of whole number, magnitude of unit digits and magnitude of decade digits) require the same response, in incompatible items the global feature ( = magnitude of whole number) does not require the same response as some of the local features ( = magnitude of unit digits). Furthermore one local feature does not require the same response as the other (decades require a different response than units). If men indeed employ a more global/holistic strategy and women a more local/decomposed strategy, we expect several interactions to arise from these manipulations which will be described in the following.

First, lateralization of activation should differ between the sexes and across the menstrual cycle. Previous imaging studies typically show right-lateralized parietal activation during number comparison (see [25] for a review) when using stimuli which do not require processing of global and local stimulus aspects (e.g. single-digit comparisons, comparison to a fixed standard). Thus, according to Fink’s hypothesis, at least in men, the local single-digit information would enter the right hemisphere, while global comparisons take place in the left hemisphere. We cautionally assume the opposite lateralization pattern in women with decreased lateralization of activation in women during the follicular as compared to the luteal phase. Furthermore, sex differences in lateralization indices should be modulated by decade crossing due to an enhanced processing effort (unit digits) for WD items when operating at the local level. We expect the decade crossing effect (WD>non-WD-items) in RT and BOLD-response in parietal areasto be stronger in women compared to men.

Second, the compatibility effect (incompatible>compatible items) in behavioural measures and BOLD-response should differ substantially between the sexes and across different cycle phases. When relying on global/holistic comparisons, performance should not differ significantly between compatible and incompatible items. Local/decomposed comparisons however require the suppression of unit-digit information in incompatible items. Consequently, local/decomposed comparisons should result in longer RT in incompatible compared to compatible items. Thus, we expect the compatibility effect in performance to be larger in women compared to men and during the luteal as compared to the follicular phase. In line with this assumption, the compatibility effect in RT was reduced in a number comparison study on a group of male participants (5 ms; [29]) compared to a number comparison study including males and females (31 ms; [24]). On the BOLD-response level, inhibitory processes involved in the processing of incompatible items may lead to stronger recruitment of prefrontal regions involved in cognitive control [30]. Thus, despite differences in deactivation areas, we expect a stronger BOLD response compatibility effect in prefrontal areas in women compared to men.

## Materials and Methods

### Ethics Statement

All subjects gave their informed written consent to participate in the study. All methods conform to the Code of Ethics of the World Medical Association (Declaration of Helsinki). The institutional guidelines of the University of Salzburg (Statutes of the University of Salzburg – see https://online.unisalzburg.at/plus_online/wbMitteilungsblaetter.display?pNr=98160) state in § 163 (1) that ethical approval is necessary for research on human subjects if it affects the physical or psychological integrity, the right for privacy or other important rights or interests of the subjects or their dependents. In § 163 (2) it is stated that it is the responsibility of the PI to decide, whether (1) applies to a study or not. Therefore we did not seek ethical approval for this study. Since it was non-invasive and performed on healthy adult volunteers who gave their informed consent to participate, (1) did not apply. Data was processed in anonymized/deidentified form. Upon arrival at the lab, participants were assigned a subject ID (v001, v002, etc.) which was used throughout the study.

### Participants

16 healthy young women (age: 26.57±6.01 years, 2 left handed) with a regular menstrual cycle (duration: 30.46±3.37 days) and 16 healthy young men (age: 25.14±4.35 years, 2 left handed) participated in the study. Female participants did not use oral or other hormonal contraceptives. No subject showed brain tissue abnormalities on structural MRI. Female participants were tested twice, once during their early follicular (onset of menstruation to 5 days before ovulation) and once during their mid-luteal phase (day 3 post ovulation to 5 days before onset of menstruation). Half of the subjects were in their early follicular phase and half of the subjects in their mid-luteal phase during the first scanning session. Cycle phase was determined by verbal reports (first day of last period, cycle duration based on last 3 periods) prior to testing and confirmed by commercial ovulation tests and follow-up evaluation of the onset of the next menstruation. No subject had to be excluded.

### Task

Two two-digit numbers were displayed above each other (Figure 1A). Participants had to decide, which number was larger. Although the task instruction (to identify the larger *number*) refers to the global level (number magnitudes not digit magnitudes) it does not explicitely state, which level to attend. Participants are free to choose whichever cognitive strategy they are comfortable with. The task can be performed on the global level, by comparing the magnitudes of the whole numbers, or on the local level, by comparing the magnitudes of the digits. In half of the items the upper number was larger and in the other half the lower number was larger. These items varied in their unit-decade compatibility and decade distance (see Figure 1 for examples). Items were unit-decade compatible (C) if the smaller number contained the smaller unit digit and unit-decade incompatible (I) otherwise. Decade distance was defined as the absolute distance between the decade digits of the two numbers. Decade distances from 1 to 4 were considered small (S), decade distances larger than 4 were considered large (L). Accordingly, items were assigned to one of 5 categories (Figure 1B): (i) compatible items with small decade distance (CS items), (ii) compatible items with large decade distance (CL items), (iii) incompatible items with small decade distance (IS items), (iv) incompatible items with large decade distance (IL items) and (v) within-decade items (WD items). Categories (i)-(iv) were summarized as non-WD items. In non-WD items, decade distance was non-zero. In WD items, decade-distance was zero. Numbers ranged from 21 to 98. In non-WD items all four digits were different. In WD items unit digits were different from decade digits. Unit distance was defined as the absolute distance between the unit digits of the two numbers. All unit distances were larger than 4.

### Procedure

In two consecutive sessions, female participants completed two sets of 150 number comparison stimuli adapted from Nuerk and coworkers [24]. Stimulus categories were matched for problem size, decades, units and parity within and across stimulus sets. 15 items per category were identical for the two stimulus sets. Identical items were matched with differing items for problem size, decades, units and parity within each stimulus set. Order of stimulus sets was randomized across sessions. Thus, half of the female participants completed the first stimulus set in the first session and half of the participants in the second session. Male participants completed one of these stimulus sets. Additionally, participants completed 30 control items per session, where they had to look at four pound keys instead of numbers without responding (null events, Figure 1A). Order of stimulus categories and control items was randomized for each stimulus set. Stimuli were presented using Presentation Software (version 0.71, 2009, Neurobehavioral Systems Inc., Albany, CA, USA) on an MR-compatible back-projection screen. Each item was presented for two seconds and followed by a one second inter-stimulus interval. Participants responded with their dominant hand. Error rates and average reaction times over correctly solved items were evaluated for each category.

### MRI Data Acquisition and Analysis

Functional images were acquired using a T1-weighted single shot echo planar (EPI) sequence (whole brain coverage, TE = 30 ms, TR = 2100 ms, flip angle 90°, slice thickness 3.0 mm with 0.6 mm gap, matrix 80×80, FOV 210 mm, in-plane resolution 2.6×2.6 mm) on a 3T Philips Gyroscan NT scanner (Philips Medical System Inc., Maastricht, The Netherlands). 36 transversal slices were taken oriented parallel to the AC-PC line. Furthermore, high resolution structural images were acquired with a T1-weighted 3D MPRAGE sequence (170 sagital slices, slice thickness = 1.2 mm, TE 3.3 ms, TR 6.8 ms, TI delay 854 ms, FA 8°, FOV 256×256, matrix 256×256).

SPM5 (http://www.fil.ion.ucl.ac.uk/spm) standard procedures and templates were used for analysis of functional images. The first five images of each session were discarded. Images were realigned to correct for head movements, unwarped to correct for interactions between head movements and EPI distortions [31] and slice time corrected. One male subject had to be removed from the sample due to excessive head motion. The mean functional image was coregistered to the high resolution structural image. The high resolution structural image was segmented and normalized to the MNI standard stereotactic space and the resulting parameters were used for normalisation of functional images. Afterwards, functional images were resampled to isotropic 3×3×3 mm voxels and smoothed with a 6 mm Gaussian kernel to enhance activation detection.

For statistical analysis we applied a two stage mixed effects model. In the subject-dependent fixed-effects first level analysis, each item category (CS, CL, IS, IL, WD) was modelled separately by a canonical hemodynamic response function. Data were high pass filtered with a cut-off of 128 seconds, and corrected for autocorrelation by an AR(1) model [32]. The parameter estimates of first-level contrasts were calculated in the context of a GLM. We defined first level contrasts for (i) the decade crossing effect (ii) WD items all compared to null events, (iii) non-WD items compared to null events (iv) the compatibility effect and (v) the decade distance effect. The parameter estimates reflect (i) a stronger signal change in response to WD items compared to non-WD items (CS, CL, IS, IL), (ii) a stronger signal change in response to WD items compared to null events, (iii) a stronger signal change in response to non-WD items compared to null events, (iv) a stronger signal change in response to incompatible (item categories IS, IL) compared to compatible items (item categories CS, CL) and (v) a stronger signal change in response to items with small (item categories CS, IS) compared to items with large decade distance (item categories CL, IL). The thereby obtained contrast images entered the group-based random-effects second level analysis (full or flexible factorial design). We employed a cluster-level FDR-corrected threshold of p<0.05. Primary thresholds were set at p<0.005 (uncorrected). Activation areas were defined as regions showing a significantly higher BOLD response to all number comparison items compared to null events in the participants compared. Deactivation areas were identified as regions showing a significantly lower BOLD response to all number comparison items compared to null events in the participants compared.

Furthermore, lateralization indices (LIs) were computed at no threshold using the LI toolbox [33] for each participant and scanning session for whole brain and the parietal lobe separately for the response to WD and non-WD items.

## Results

**Figure 2:**
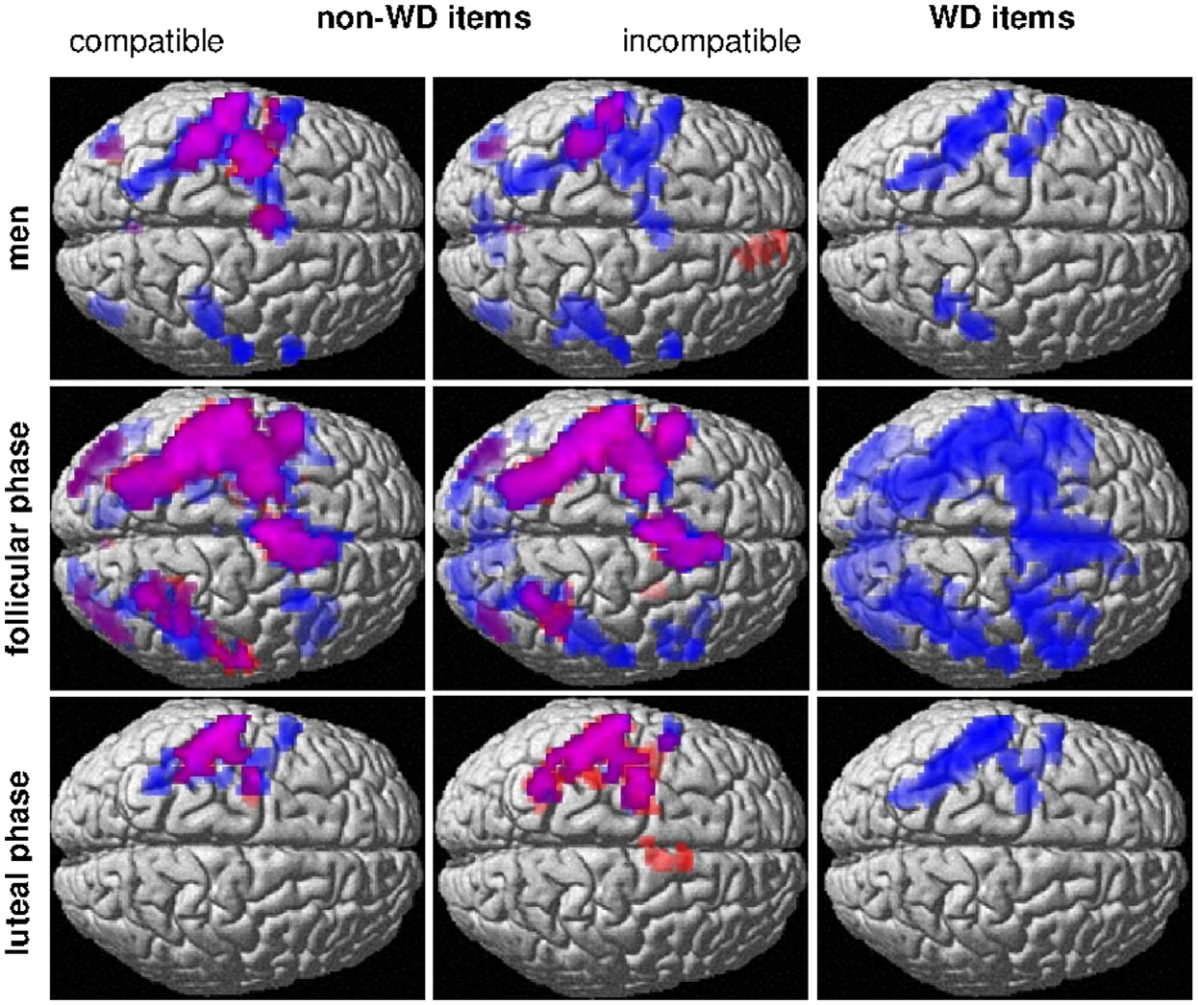
BOLD-response to number comparison for all item categories in men and women during follicular and luteal phase. WD = within-decade, Blue = small decade distance, Red = large decade distance.

The effects of decade crossing (WD vs. non-WD) and compatibility (incompatible vs. compatible) in behavioural measures and BOLD-response were strongly modulated by either sex or menstrual cycle or both and will be reported separately for each group and cycle phase below with respect to menstrual cycle dependent changes or sex differences. The effect of decade distance was not modulated by sex or menstrual cycle phase in behavioural measures or BOLD-response, did not interact with compatibility and was therefore not analysed further. In men as well as women and during both cycle phases, we found higher RTs and ERs (RTs: all F >104.11, all p<0.001; ERs: all F >6.80; all p<0.05), as well as stronger BOLD-response in task-related areas (compare Figure 2) for items with small decade distance compared to items with large decade distance. Due to the observed menstrual cycle-dependent changes, we decided to assess sex differences separately for the follicular and luteal cycle-phase. Although the menstrual cycle design was counterbalanced, we used scanning session as a covariate in all sex-comparisons to account for possible learning effects.

### Behavioural Results

#### Menstrual cycle dependent changes

While menstrual cycle did not affect RT in non-WD items (t_(13)_ = 1.36, p = 0.20), women responded significantly faster to WD items in their luteal phase as compared to their follicular phase (t_(13)_ = 3.44, p = 0.004). There were no significant menstrual cycle dependent changes in ER in WD or non-WD items.

**Figure 3:**
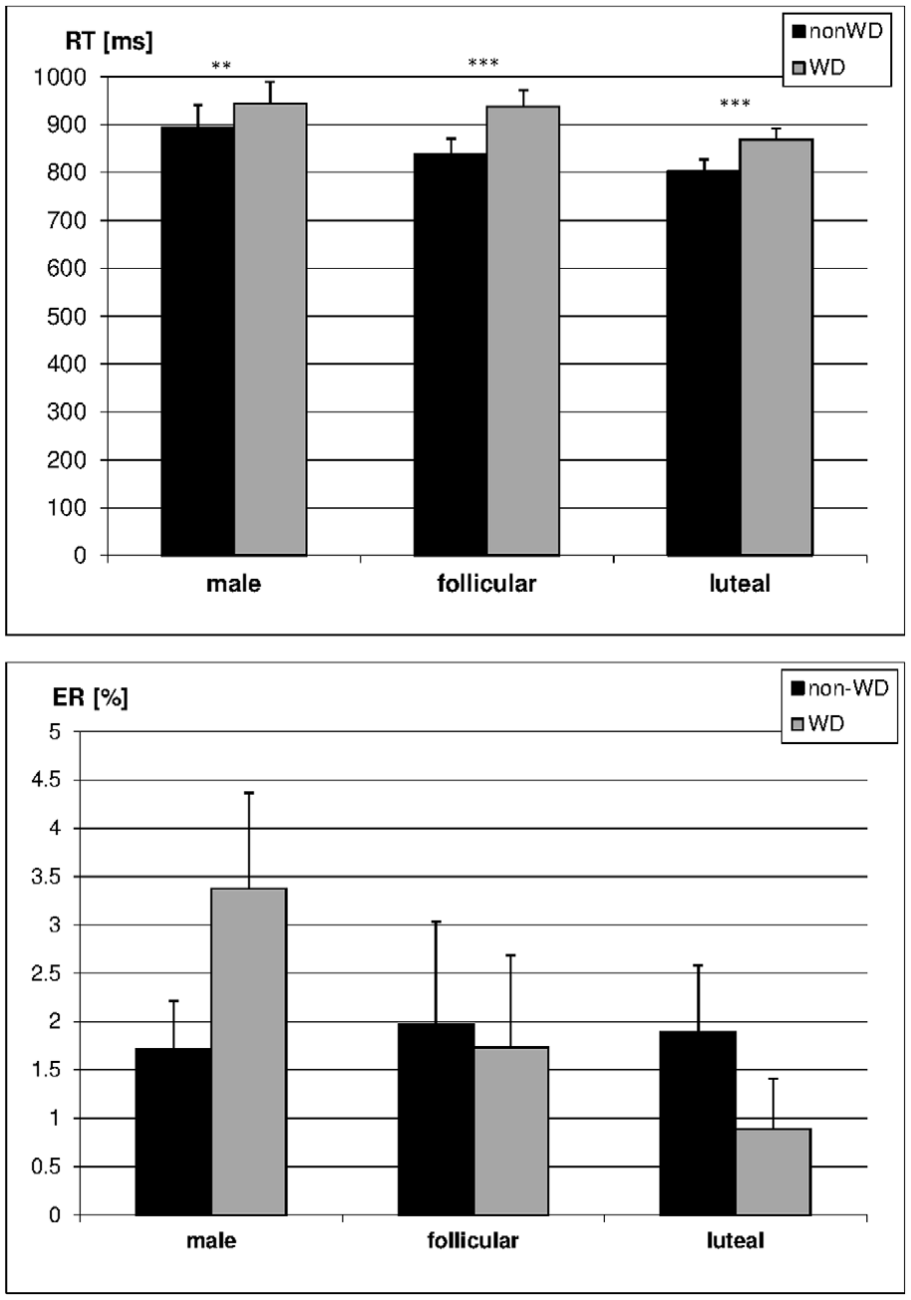
Decade crossing effect in reaction times (RT) and error rates (ER) in men and women during different cycle phases. Responses to within-decade (WD) items were significantly slower than responses to non-WD items in all participants. The effect was significantly stronger in women during their follicular phase compared to their luteal phase and men.

A significant *decade crossing effect* in RT was found during both cycle phases with reactions to WD items being slower compared to reactions in non-WD items (both t_(14)_ >5.18, both p<0.001, compare Figure 3A). A 2×2-ANOVA with decade crossing (WD vs. non-WD) and cycle phase (follicular vs. luteal) as within-subjects factors revealed that the effect of decade crossing in RT was significantly smaller during the luteal phase as compared to the follicular phase (F_(1,13)_ = 6.21, p<0.05). There were no significant menstrual cycle dependent changes in the decade crossing effect in ER (Figure 3B).

**Figure 4:**
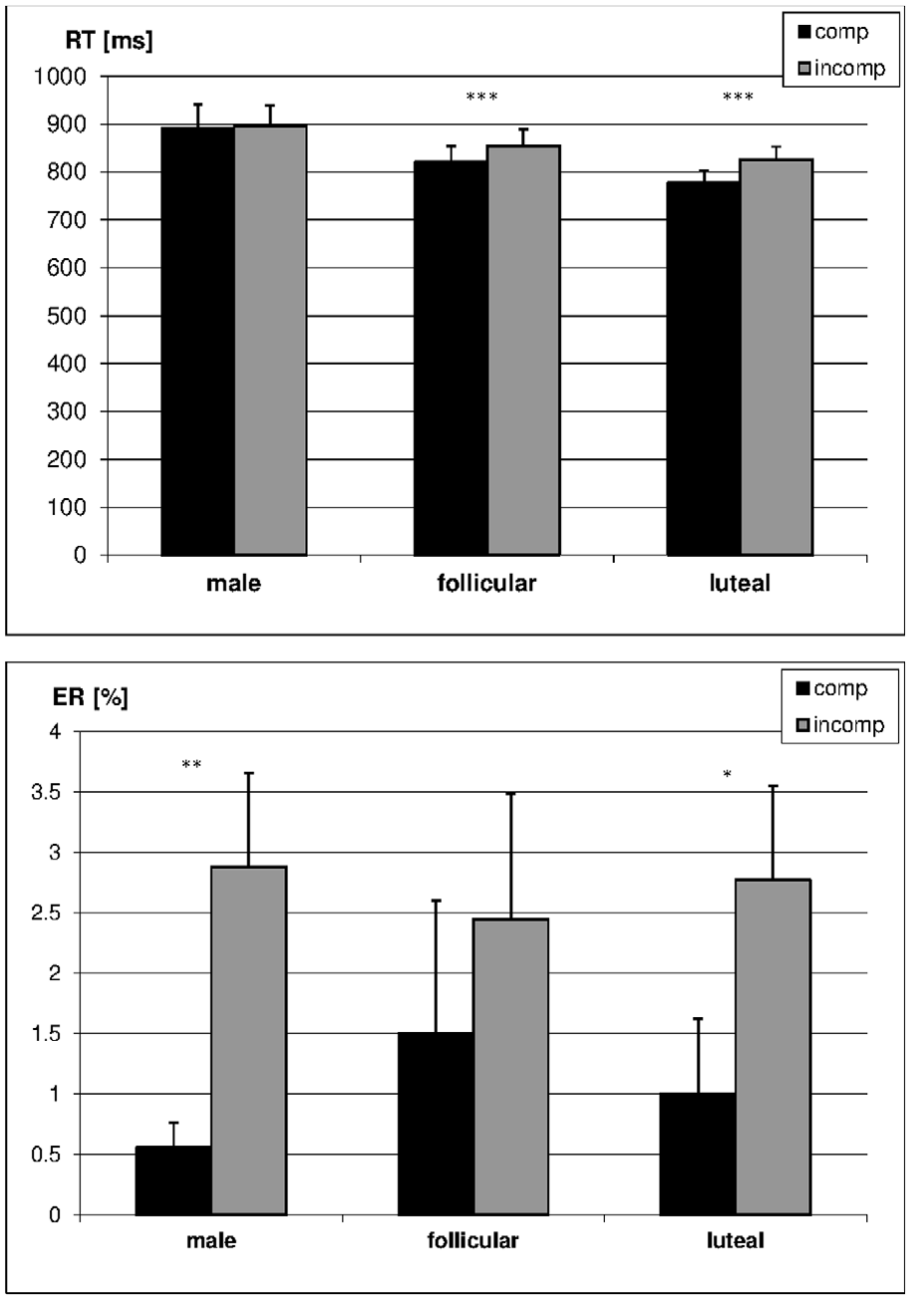
Compatibility effect in reaction times (RT) and error rates (ER) in men and women during different cycle phases. Responses to incompatible items were significantly slower than responses to compatible items in women, but not in men.

Furthermore a significant *compatibility effect* in behavioural measures was observed during both cycle phases. Women showed significantly higher RTs (follicular phase: t_(14)_ = 4.52, p<0.001; luteal phase: t_(14)_ = 5.06, p<0.001, Figure 4A) and by trend higher ERs (follicular phase: t_(14)_ = 1.93, p = 0.07, luteal phase: t_(14)_ = 2.11, p = 0.05, Figure 3B) in incompatible compared to compatible items. There were no significant menstrual-cycle dependent changes in the compatibility effect in behavioural measures as revealed by 2×2 ANOVAs on RT and ER with compatibility and cycle phase as within-subjects factors (F_(1,13)_ = 0.39, p = 0.55).

#### Sex differences

Women during luteal phase responded significantly faster to non-WD items (F_(1,27)_ = 5.27, p<0.05) and by trend faster to WD items (F_(1,27)_ = 3.41, p = 0.08) than men, with RTs of women during follicular phase lying in-between (compare Figure 3). There were no significant sex differences in ER in WD or non-WD items.

As women, men showed a significant *decade crossing effect* in RT by responding significantly slower to WD-items than to non-WD items (t_(14)_ = 3.78, p<0.01, compare Figure 3A). Two separate 2×2 ANCOVAs with decade crossing as within-subjects factor, sex as between-subjects factor and scanning session as a covariate revealed that the effect of decade crossing was significantly smaller in men compared to women during their follicular phase (F_(1,27)_ = 4.37, p<0.05), but did not differ between men and women when they were in their luteal phase (F_(1,27)_ = 1.47, p = 0.24). There were no significant sex differences in the decade crossing effect in ER (all F_(1,27)_ <0.71, all p>0.38, Figure 3B).

Other than women, men did not show a *compatibility effect* in RT (t_(14)_ = −0.34, p = 0.74, Figure 4A). Two separate 2×2 ANCOVAs with compatibility as within-subjects factor, sex as between-subjects factor and scanning session as a covariate showed that sex interacted significantly with the compatibility effect in RT during both cycle phases (follicular phase: F_(1,27)_ = 3.18, p = 0.08; luteal phase: F_(1,27)_ = 8.72, p<0.01). However, men did show a compatibility effect in ER (t_(14)_ = 3.13, p<0.01, Figure 4B) which was by trend larger compared to women when they were in their follicular phase (F_(1,27)_ = 3.79, p = 0.06), but not when they were in their luteal phase (F_(1,27)_ = 0.97, p = 0.33). To account for possible speed accuracy trade-offs (Table 1), average RT was entered as a second covariate in the above ANCOVAs. This reverses the sex difference in the compatibility effect, such that women during their follicular phase show by trend a stronger compatibility effect compared to men (F_(1,27)_ = 3.93, p = 0.06). When introducing RT as a covariate in the comparison of ER in compatible and incompatible items, the ER compatibility effect in men diminished, while the compatibility effect in women during their follicular phase was enhanced. There was no speed-accuracy trade-off in compatible or incompatible items over all participants (all r <0.11, all p>0.47).

**Table 1:** Speed accuracy trade-offs.

	Correlation coefficients for ER with average RT				Size of Compatibility effect in ER	
	Comp.	Incomp.	WD	Comp. effect^1^	without cov. RT	with cov. RT
Men	−0.16	0.25	−0.64* 2	0.28	F = 9.78**	F = 0.22
Follicular	0.22	−0.05	−0.06	−0.59* 4	F = 3.74∼	F = 8.64**
Luteal	−0.58* 3	0.15	−0.31	0.39	F = 4.45*	viol.

Correlations of average reaction time (RT) with error rate (ER) in different item categories and with the ER compatibility effect, as well as strength of the compatibility effect before and after controlling for RT in an ANCOVA with compatibility as within-subjects factor.

Comp. = compatible items, Incomp. = incompatible items, WD = within-decade items, viol. = ANCOVA conditions violated,

∼ p<0.1,

* p<0.05,

** p<0.01.

1 Compatibility effect in ER = (ER in incompatible items – ER in compatible items).

2 this correlation is by trend stronger than in women during their follicular phase (Z = −1.78, p = 0.08) and significantly larger than the correlations with ER in compatible and incompatible items (both Z >7.73, p<0.001).

3 this correlation is significantly stronger than during the follicular phase (Z = 2.26, p<0.05) and significantly larger than the correlations with ER in incompatible and WD items (both Z >4.43, p<0.001).

4 this correlation is significantly stronger than during the luteal phase and in men (both Z >2.26, both p<0.01).

### Neuroimaging Results

Independent of sex, cycle phase or item category the number comparison task activated a largely left lateralized (non-WD: total LI = 0.08±0.09, parietal LI = 0.25±0.18; WD: total LI = 0.06±0.08, parietal LI = 0.20±0.18; all t_(44)_ >5.08, all p>0.001) network including the pre- and postcentral gyri, inferior and superior parietal lobules and lateral frontal areas. Figure 2 gives an overview of the hemodynamic response patterns to each item category in men and women during their follicular and during their luteal cycle phase.

#### Menstrual cycle dependent changes

**Figure 5:**
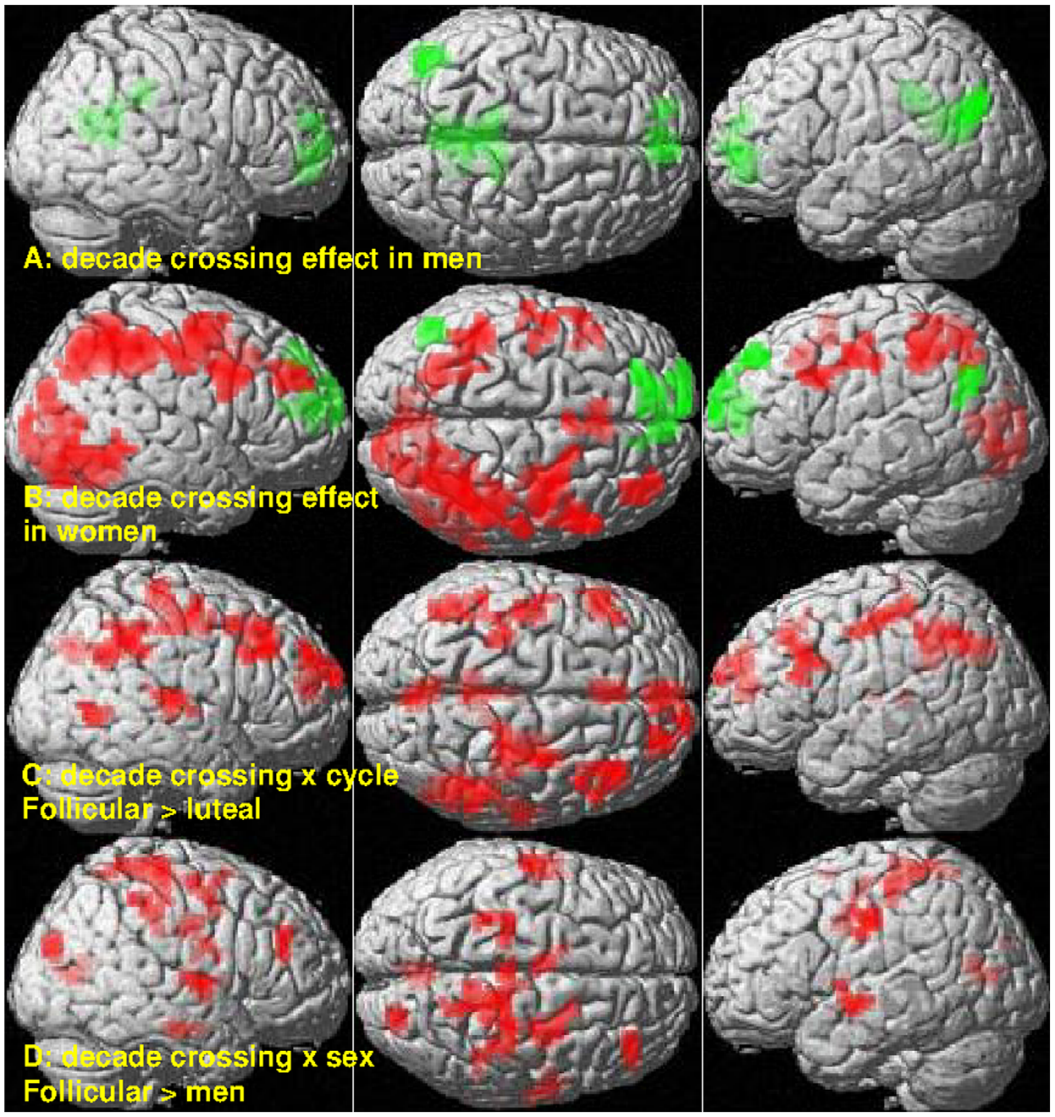
Sex- and menstrual cycle-dependent differences in the decade crossing effect in BOLD-response. A. Decade crossing effect in men: deactivation was stronger in within-decade (WD) compared to non-WD items (green). B. Decade crossing effect in women: During the follicular phase fronto-parietal activation was stronger in WD compared to non-WD items (red), particularly in the right hemisphere. During the luteal phase deactivation was stronger in WD compared to non-WD items (green). C. Menstrual cycle dependent effects: The decade crossing effect in activation areas was stronger during the follicular phase compared to the luteal phase. D. Sex differences: The decade crossing effect in activation areas was stronger in women during the follicular phase compared to men.

Lateralization indices per se did not differ across the menstrual cycle (all |t_(15)_| <0.87, all p>0.40). However, while women were in their follicular phase activation in response to WD items was more bilateral than to non-WD items (total LI: t_(15)_ = 2.21, p<0.05, parietal LI: t_(15)_ = 3.74, p<0.01), but there were no significant differences in lateralization indices between WD and non-WD items during the luteal phase (total LI: t_(15)_ = 0.39, p = 0.37, parietal LI: t_(15)_ = 0.56, p = 0.59). Thus, the effect of decade crossing on lateralization indices was by trend stronger during the follicular phase than during the luteal phase (F_(1,15)_ = 4.16, p = 0.06). Therefore, during the follicular phase, BOLD-response to WD items was significantly stronger compared to non-WD items in right hemispheric areas corresponding to the left-hemispheric activation areas (Figure 5A). During the luteal phase no significant activation differences were present between WD and non-WD items. However, during the luteal phase, there was slightly stronger deactivation in regions of the default mode network (Figure 5A) in WD items compared to non-WD items. Consequently, the effect of decade crossing in women was significantly larger in several right hemispheric areas and significantly smaller in DMN regions during their follicular phase compared to their luteal phase (Figure 5B, Table 2).

**Table 2:** Clusters with significant menstrual cycle dependent modulation or sex differences in the decade crossing effect in BOLD-response (p<0.05, FDR-corrected at cluster-level).

Brain region	MNI-coordinates (mm)			Side	#voxels	*T*	*p_FDR_*
	X	Y	Z				
**Menstrual cycle dependent changes (follicular>luteal)**							
Precentral/postcentral	33	−12	51	right	126	6.58	<0.001
Postcentral/precentral	−42	−9	48	left	40	5.21	0.009
Postcentral/precentral	−30	−33	60	left	28	5.06	0.028
IPL	54	−45	39	right	121	4.88	<0.001
IPL	−45	−57	45	Left	62	4.54	0.001
mPFC	15	66	21	right	104	5.61	<0.001
mPFC	6	54	39		24	3.82	0.046
mPFC	−6	24	45		32	3.62	0.018
Precuneus	−3	−72	36		53	3.75	0.003
Posterior cingulate g.	−3	−36	30		67	4.72	0.001
Middle temporal	45	−57	3	right	36	4.59	0.012
Superior temporal g.	60	−24	9	right	39	4.95	0.009
Superior frontal	18	36	51	right	23	4.22	0.050
Superior/middle frontal	21	51	30	right	35	4.64	0.013
Middle frontal	39	30	36	right	87	4.56	<0.001
Inferior frontal	−48	18	30	Left	50	4.12	0.003
**Sex differences (follicular>men)**							
Precentral/postcentral	15	−33	69	Right	116	4.01	<0.001
Postcentral/IPL	36	−33	48	right	41	4.02	0.045
Precentral/postcentral	−27	−24	57	Left	46	3.87	0.045
Postcentral/precentral	−60	−15	39	Left	32	4.89	0.046
SMA	0	−27	54		34	4.42	0.046
Middle cingulate g.	−6	−6	42		33	4.20	0.046
Hippocampus/parahippocampus	27	−18	−18	right	33	5.21	0.046
Calcarine g.	0	−66	15		31	3.68	0.046
Superior/middle frontal	33	0	48	right	39	3.90	0.045
Middle frontal	36	36	27	right	29	4.66	0.050
Lateral occipital g.	24	−84	24	right	32	4.45	0.046
Superior temporal g.	60	−6	6	right	29	4.39	0.050
Superior temporal g.	−54	−6	−6	Left	31	4.11	0.046

g. = gyrus, SMA = supplementary motor area, mPFC = medial prefrontal cortex, IPL = inferior parietal lobule.

**Figure 6:**
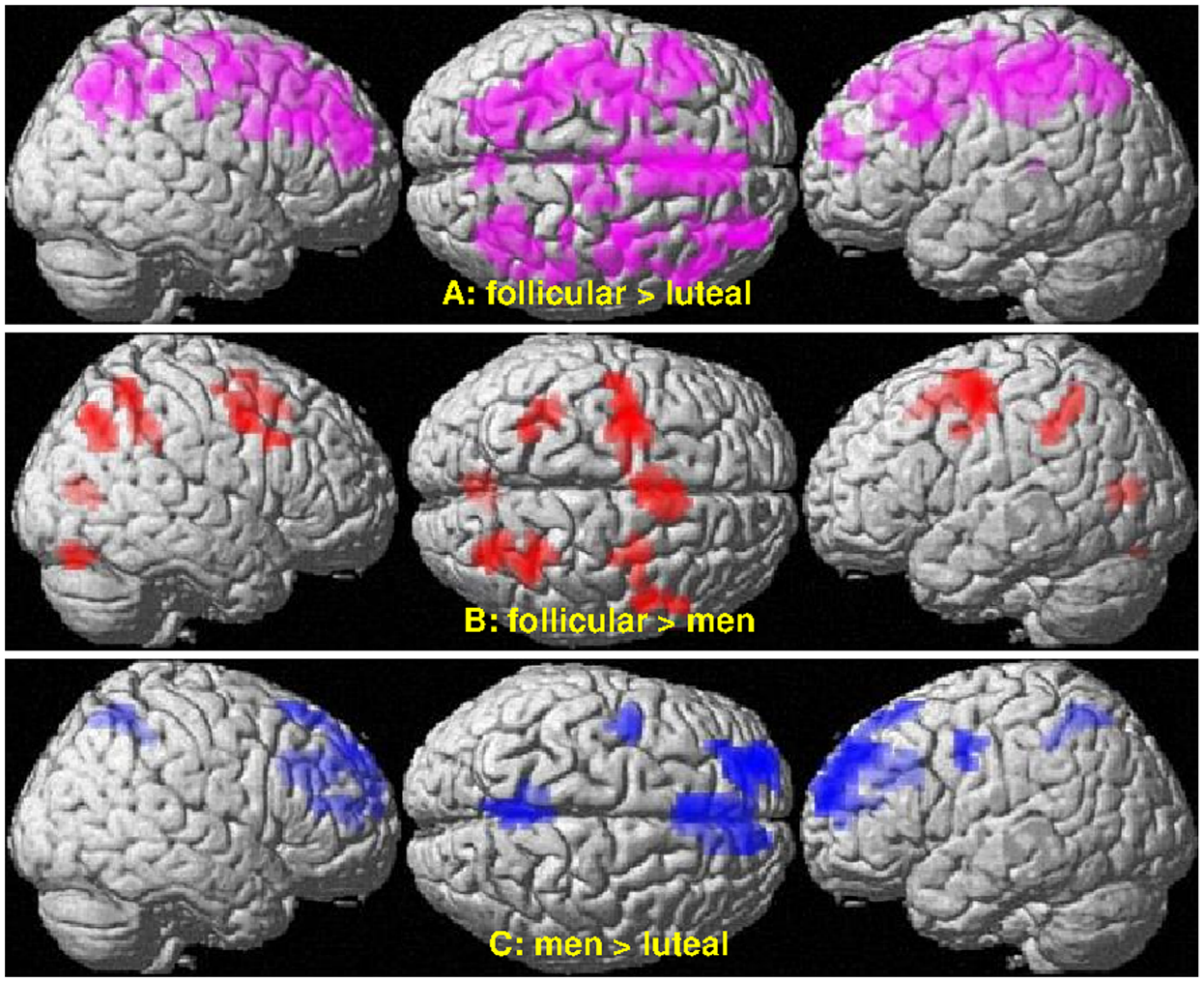
Sex- and menstrual cycle-dependent differences in the BOLD-response to WD items. Displayed are clusters with significantly higher BOLD-response (p<0.05, FDR-corrected at cluster level) in women during their follicular phase compared to (A) their luteal phase and (B) men, as well as (C) higher BOLD-response in men compared to women during their luteal phase. See Results and Table 1 for cluster labels.

During follicular phase, women showed significantly stronger BOLD-response to WD items than during luteal phase in a large fronto-parietal network and in midline deactivation areas (Figure 6A, Table 3). BOLD-response variations across the menstrual cycle were not significant in non-WD items.

**Table 3:** Clusters with A. significant menstrual cycle dependent modulation or B. significant sex differences in the BOLD-response (p<0.05, FDR-corrected at cluster-level) to within decade (WD) items.

Brain area	MNI-coordinates (mm)			Side	#voxels	*T*	*p_FDR_*
	X	Y	Z				
**Menstrual cycle dependent changes (follicular>luteal)**							
Inferior/middle frontal g.	−36	18	33	Left	150	6.28	<0.001
Superior/middle frontal g.	−21	48	15	Left	70	6.10	<0.001
Middle/superior frontal g.	33	54	18	Right	108	5.13	<0.001
Middle/superior frontal g.	33	27	33	Right	27	3.75	0.026
Middle/inferior frontal g.	51	18	30	Right	64	5.00	<0.001
SMA/mPFC	6	15	39		478	5.94	<0.001
Para−/precentral g./SMA	18	−21	66	Right	47	4.93	0.003
Pre−/postcentral g.	33	−6	54	Right	103	6.53	<0.001
Postcentral g./inf. parietal g.	48	−33	54	Right	42	4.53	0.005
Parietal lobe	30	−66	42	Right	164	5.34	<0.001
Pre−/postcentral g./parietal lobe	−33	−6	54	Left	664	5.77	<0.001
Posterior cingulate g.	−6	−36	9		31	4.76	0.018
Precuneus	6	−69	51		29	4.30	0.021
**Sex differences**							
**(a) follicular>men**							
Superior/middle frontal g./precentral g.	−30	−3	63	Left	168	5.39	<0.001
Precentral g./superior/middle frontal g.	30	−3	51	Right	35	4.39	0.044
Precentral g./inferior/middle frontal g.	54	0	42	Right	36	3.70	0.044
SMA	0	15	51		103	4.43	<0.001
Superior parietal lobule	−33	−42	39	Left	61	4.94	0.006
Superior parietal lobule	27	−42	39	Right	78	4.44	0.002
Superior parietal lobule/middle occipital g.	30	−72	45	Right	89	6.95	0.001
Calcarine g./lingual g.	−3	−75	9		46	4.23	0.021
Cerebellum	30	−78	−21	Right	36	4.07	0.044
**(b) men>luteal**							
Superior/middle frontal	−24	57	18	Left	185	5.43	<0.001
mPFC	−6	24	60		83	5.16	0.001
Middle/anterior cingulate gyrus/mPFC	−6	39	36		128	4.96	<0.001
Anterior cingulate gyrus/mPFC	15	36	18	Right	52	4.04	0.011
Precentral g.	−42	−3	45	Left	38	4.79	0.036
Precuneus	−3	−51	54		76	5.75	0.002

Refer to the main text for clusters with significant menstrual cycle dependent modulation or sex differences in the BOLD-response to non-WD items. g. = gyrus, SMA = supplementary motor area, mPFC = medial prefrontal cortex.

**Figure 7:**
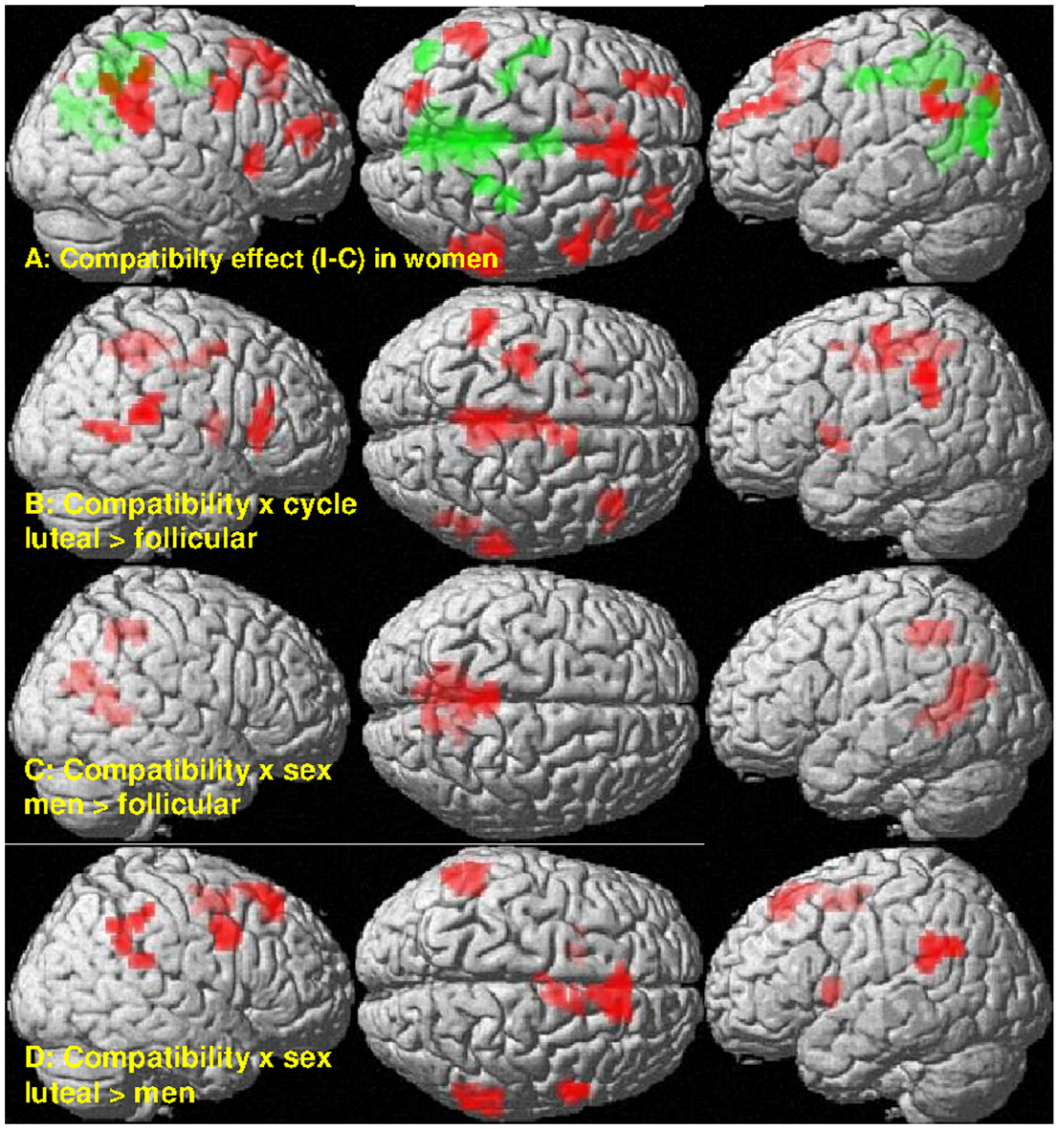
Sex- and menstrual cycle-dependent differences in the BOLD-response compatibility effect. A: Women during follicular phase show higher BOLD-response for compatible compared to incompatible items (negative effect of compatibility) in deactivation areas (green). Thus, deactivation is stronger for incompatible compared to compatible items. Women during luteal phase show higher BOLD-response for incompatible compared to compatible items (positive effect of compatibility) in the frontal cortex (red). Men did not show a significant compatibility effect. B-D: Consequently compatibility x sex/cycle interactions were observed in frontal and deactivation areas (see Table 2 for cluster labelling).

Women also showed BOLD-response differences between compatible and incompatible items, with location and direction of the effect depending on their menstrual cycle phase (Figure 7A). When women were in their follicular phase they showed significantly stronger BOLD-response, i.e. less deactivation, for compatible compared to incompatible items (negative effect of compatibility) in several more posterior areas, attributed to the default mode network, including the precuneus, posterior cingulate gyrus, and bilateral inferior parietal lobules. Contrarily, when they were in their luteal phase, they showed significantly stronger BOLD response for incompatible compared to compatible items (positive effect of compatibility) in several more anterior areas, including the supplementary motor area and lateral prefrontal cortices (i.e. more activation), as well as the bilateral inferior parietal lobules (i.e. less deactivation). Consequently, the compatibility effect interacted significantly with menstrual cycle phase in these areas (Figure 7B, Table 4).

**Table 4:** Clusters with significant menstrual cycle dependent modulation or sex differences in the compatibility effect (incompatible>compatible) in BOLD-response (p<0.05, FDR-corrected at cluster-level).

Brain region	MNI-coordinates (mm)			Side	#voxels	*T*	*p_FDR_*
	X	Y	Z				
**Menstrual cycle dependent changes (luteal>follicular)**							
Frontal triangular gyrus	39	27	6	Right	50	6.37	0.003
Precentral gyrus	−27	−21	57	Left	47	4.32	0.004
Inferior parietal lobule	−48	−42	33	Left	70	5.69	0.001
Posterior cingulate gyrus/precuneus	6	−36	42		108	4.57	<0.001
Anterior cingulate gyrus	9	6	51		61	4.63	0.001
Superior temporal gyrus	63	−33	15	Right	37	4.83	0.012
Middle temporal gyrus	51	−54	6	Right	25	4.06	0.042
Putamen	−33	3	−3	Left	25	4.43	0.042
Pallidum/caudate nucleus	15	0	3	Right	27	4.12	0.040
**Sex differences**							
**(a) follicular>men**							
Calcarine g./Precuneus/Cuneus	9	−48	3		353	5.59	<0.001
Precuneus/middle cingulate g.	−6	−39	51		110	5.12	<0.001
**(b) men>luteal**							
Precentral g.	57	6	39	Right	36	4.80	0.039
SMA/superior frontal g.	6	−3	54		43	4.71	0.024
mPFC/SMA	9	30	51		106	4.52	<0.001
IPL	−57	−57	30	Left	73	4.40	0.003
Putamen/Caudate	−24	6	6	Left	34	4.38	0.041
IPL	63	−45	30	Right	48	3.71	0.019

g. = gyrus, SMA = supplementary motor area, mPFC = medial prefrontal cortex.

#### Sex differences

There were no sex differences in lateralization indices per se (all |t_(27)_| <0.95, all p>0.35).

As women during their luteal phase, men showed no decade crossing effect in activation areas, but stronger deactivation for WD items compared to non-WD items in default mode network regions (Figure 5A). Thus, there were no sex differences present in the decade crossing effect in BOLD-response and lateralization indices, when women were in their luteal phase. However during follicular phase, BOLD-response to WD items was less lateralized than to non-WD items in women compared to men (F_(1,27)_ = 4.26, p<0.05). Consequently, the effect of decade crossing was significantly larger in women during follicular phase as compared to men in the right postcentral/superior parietal gyri (Figure 5C, Table 2).

**Figure 8:**
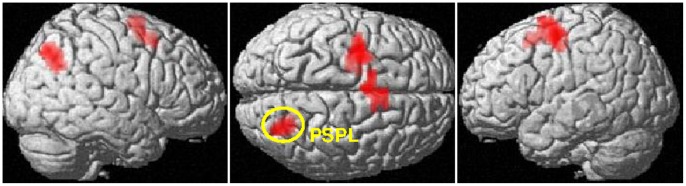
Differential activation of the PSPL between men and women. In response to non-WD items, women during their follicular phase show stronger BOLD-response in the posterior superior parietal lobule (PSPL; [30, −69, 42]) compared to men, as well as stronger BOLD-response in the supplementary motor area and left superior frontal gyrus.

During follicular phase, women showed significantly stronger BOLD-response compared to men to WD items in a large bilateral fronto-parietal network (Figure 6B, Table 3) and to non-WD items in the right PSPL ([30, −69, 42], T_(27)_ = 7.58, p_FDR_ = 0.001, k = 83), as well as the supplementary motor area ([−3, 0, 63], T_(27)_ = 4.87, p_FDR_ = 0.001, k = 82) and left superior frontal gyrus ([−27, −6, 54], T_(27)_ = 4.79, p_FDR_ <0.001, k = 111) (Figure 8). There were no differences between men and women during follicular phase in deactivation areas.

During luteal phase, women showed significantly weaker BOLD-response compared to men to WD items in some task-related activation areas, like the left pre- and postcentral gyri, the superior parietal lobules and lateral frontal areas (Figure 6C, Table 3) as well as classical default mode network regions, like the precuneus, anterior cingulate gyrus and medial prefrontal cortex. Thus, in the respective areas activation was weaker and deactivation was stronger in women during luteal phase compared to men. BOLD-response to non-WD items did not differ significantly between men and women during luteal phase.

Unlike women, men did not show a compatibility effect in BOLD-response. Consequently, women showed a significantly stronger compatibility effect than men in the precuneus, when in their follicular phase and in the mPFC and IPLs when in their luteal phase (Figure 7C-D, Table 4).

## Discussion

The present study was designed to investigate, whether sex and/or menstrual cycle phase affect whether multi-digit numbers are processed in a more global/holistic or local/decomposed fashion. We employed a number comparison task in which participants had to decide which of two vertically aligned numbers was larger. Some items were compatible (e.g. 23 vs. 67), i.e. the smaller number contained the smaller unit digit, some were incompatible (e.g. 27 vs. 63), i.e. the smaller number contained the larger unit digit and some were WD items (e.g. 63 vs. 67), i.e. they differed only in their unit digit (compare Figure 1). We proposed a more global/holistic processing strategy of whole number magnitudes in men and a more local/decomposed processing strategy of digit magnitudes in women. Furthermore we proposed stronger global/holistic processing in women during the follicular as compared to the luteal cycle phase. To test this hypothesis, we were interested in sex-differences and menstrual cycle modulation of hemispheric asymmetries on the one hand and the decade crossing and compatibility effects in RT and BOLD-response on the other hand. The sex and menstrual cycle dependent interactions with the decade crossing and compatibility effect observed in RT were reflected on the BOLD-response level in DMN deactivation. It has been demonstrated that the challenge of a task [34] and RT [35] relates to the strength of deactivation within the default mode network (DMN; [36]–[37]). Therefore, we will base our following discussion mainly on the differences we observed in activation areas.

It is per se interesting, that parietal activation in this study is stronger in the left hemisphere, while a stronger right hemispheric response to number comparison has been reported in previous studies [38]–[40]. However, these studies used either single-digit comparisons or comparison to a fixed standard, for which simultaneous processing of global and local level are not required, while the present study presented the numbers to compare simultaneously above each other. Interestingly also, there was no main effect of sex and menstrual cycle phase on lateralization indices. Activation was left lateralized in both men and women and during both cycle phases. While this result is contrary to previous results from emotional memory tasks showing opposite lateralization patterns in men and women [14], it does not necessarily contradict the model of more global/holistic processing in men and more local/decomposed processing in women. We predicted left-hemispheric processing of global information in men due to Fink’s model that (male) hemisphere’s are by default in global mode and that local information is more challenging and enters the hemisphere specialized for the content. If women’s hemisphere’s are not by default in global mode, but in local mode and global information is more challenging, we would expect global information to enter the hemisphere specialized for the content, i.e. the right hemisphere in this case, while local information would be processed in the left hemisphere. Thus, left lateralized processing could mean global processing in men, but local processing in women. Admittedly, there are a lot of assumptions to that explanation, which require further investigation. However, our data fit all the other predictions drawn from the model of more global/holistic processing in men and local/decomposed processing in women.

As expected, sex interacted significantly with the decade crossing effect behaviourally and in terms of lateralization. The decade crossing effect was significantly stronger and processing of WD as compared to non-WD items more bilateral in women during the follicular phase as compared to men. During the luteal phase however lateralization indices do not differ between WD and non-WD items and the behavioural decade crossing effect is not stronger than in men. This menstrual cycle dependent effect supports the view of stronger global processing during the follicular phase on the one hand and is in line with previous works demonstrating reduced interhemispheric inhibition and functional cerebral asymmetries during the low-progesterone follicular phase on the other hand [21]–[22], [41].

There are two ways to compare numbers at the local/decomposed level and these data may demonstrate a shift between strategies over the menstrual cycle. The most analytic way is to compare decade-digits and only in case they do not give enough information, also compare the unit-digits to reach a conclusion. However, since large digits are more salient and draw attention automatically [42], an alternative strategy is to just watch out for the largest digits (large digit heuristic). This pattern obviously requires less cognitive control than the analytic strategy. It has been demonstrated previously that higher levels of estrogen, as observed during the luteal phase, are associated with less efficient inhibitory control [43]–[45]. Thus, presumably, if operating at the local level, women should be more likely to base their decisions on a heuristic in the luteal phase, while during the follicular phase they are able to analytically compare decade and unit digits one after the other. For WD items, decades are not distinguishable, but the larger number still contains the larger digits. Consequently, additional processing of either the global level or the unit digits is necessary during the follicular phase but not during the luteal phase. Since parietal activation to WD items is stronger in both hemispheres during the follicular phase as compared the luteal phase and men, it may be the case that they are processing local and global information in parallel during those items where decade digit information is inconclusive. Further supporting this view, women show stronger BOLD-response in the right PSPL than men during their follicular phase. The PSPL is involved in attentional orientation on the mental number line [25] and more active when performing two operations as compared to one [46].

However, the strongest support for the hypothesis of global/holistic processing in men and local/decomposed processing in women comes from the compatibility effect. While men lack a compatibility effect in RTs as well as BOLD-response, women show longer RTs to incompatible compared to compatible items as well as significant BOLD-response modulation by compatibility during both cycle phases. During their follicular phase, women show stronger deactivation of DMN regions for incompatible compared to compatible items, while no differences were observed in activation areas. During their luteal phase, women show stronger activation of prefrontal areas for incompatible compared to compatible items. Comparison of incompatible items should not differ from the comparison of compatible items on the global level, but it requires the suppression of unit digits on the local level. The latter is especially true when employing a large digit heuristic as we assume for women during the luteal phase. The recruitment of prefrontal areas in incompatible items during the luteal phase is in line with the assumption of additional inhibitory control processes being involved in the processing of incompatible items at the local level (see [30] for a review of cognitive control and prefrontal cortex functioning). With the more analytic local strategy as assumed for the follicular phase this suppression of unit digits should take place in all non-WD items. This is supported by the sex difference in the BOLD-response in frontal areas during non-WD, demonstrating stronger activation in women during follicular phase as compared to men. Therefore the compatibility effect is restricted to DMN areas during the follicular phase and not observed in prefrontal areas. Furthermore, a decrease in inhibitory control during the luteal phase [42]–[44] may also lead to stronger recruitment of prefrontal areas.

In summary, our data support the view of a stronger focus on the global level (holistic processing) in men and a stronger focus on the local level (decomposed processing) in women. Furthermore, women’s processing is more bilateral and appears to be more analytic/controlled during the follicular as compared to the luteal phase. Our data outline that lateralization plays an important role in the processing of global and local information, but they also suggest that stimulus content, sex and menstrual cycle phase need to be taken into account to establish a model of hemispheric asymmetries in global and local processing.
